# Evaluation of patient setup uncertainty of optical guided frameless system for intracranial stereotactic radiosurgery

**DOI:** 10.1120/jacmp.v11i2.3181

**Published:** 2010-04-17

**Authors:** Jia‐Zhu Wang, Roger Rice, Todd Pawlicki, Arno J. Mundt, Ajay Sandhu, Joshua Lawson, Kevin T. Murphy

**Affiliations:** ^1^ Department of Radiation Oncology, Moores Cancer Center University of California San Diego, La Jolla CA USA

**Keywords:** stereotactic radiosurgery, linac‐based radiosurgery, frameless localization, patient setup uncertainty

## Abstract

The optically‐guided frameless system (OFLS) has been used in our clinic for intracranial stereotactic radiosurgery (SRS) since 2006, as it is especially effective in IMRT‐based radiosurgery (IMRS), which allows treating multiple brain lesions simultaneously using single isocenter approach. This study reports our retrospective analysis of patient setup accuracy using this system. The OFLS consists of a bite block with fiducial markers and an infra‐red camera system. To test reproducibility, patients are taken for reseat verification after bite block construction. Upon the completion of radiosurgery planning, the isocenter position(s) and images are sent to the optical guidance computer where fiducials are manually registered from the CT scan. During treatment, patient setup is monitored and guided by the camera readings on the fiducials. In addition, two orthogonal kV images are acquired and used as an isocenter verification tool. In addition, we have analyzed the reseat and fiducial digitization data of 56 patients. Retrospective comparison of kV images with reference images has been carried out for all the patients to evaluate actual patient setup accuracy at the time of treatment. The histogram of the findings shows that 82.2% of patients had 3D isodisplacement (E≤1mm; 5.2% had 1<E≤2mm). Hence, for 87.5 % of the patients in the study, treatments were finished under the optical guidance with a maximum setup error of 2 mm and the median setup error of 0 mm. For the remaining 12.5% of patients in the study, the isodisplacements were greater than 2 mm and the treatment records showed that those patients were repositioned, guided by the orthogonal kV‐images. It is found that the OFLS in the SRS treatment has acceptable accuracy when used in conjunction with orthogonal kV images, and the use of orthogonal kV images as a verification tool ensures the efficacy of frameless localization in the radiosurgery treatment.

PACS numbers: 87.53.Ly, 87.61.Tg, 87.55.Qr, 87.56.‐v, 29.20.Ej

## I. INTRODUCTION

Linac‐based SRS is used to treat both malignant and benign brain lesions. The radiation delivery has traditionally been done in terms of non‐coplanar rotational arc beams, with the aid of circular cones to provide beam collimation. This technique is still being used in many clinics.^(^ ^1^ ^–^ ^6^ ^)^

To achieve treatment accuracy, a stereotactic head frame is routinely used for patient immobilization and target localization. A rigid head frame, such as the Brown‐Roberts‐Wells (BRW) head ring, uses four pins screwed into the skull and attached either to a floor‐mount or to a couch‐mount system. This invasive procedure is time consuming and painful. Patients normally wear the frame for several hours and wait to be treated later in the day. The treatment time for each lesion is approximate 50–60 minutes. The advent of intensity modulated radiation treatment (IMRT) with dynamic multileaf collimators (MLC) provides optimal coverage for tumor volume and minimizes the dose to adjacent critical organs. This technique has been implemented for intracranial SRS in many institutions, including our own.^(^ ^7^ ^–^ ^15^ ^)^

We have been using the cone‐based arc beams for stereotactic radiosurgery (SRS) to treat small solitary lesion(s), and the dynamic MLC‐based fixed gantry beams for intensity modulated stereotactic radiosurgery (IMRS) to treat multiple brain metastases or larger irregular brain tumor(s). The IMRS approach enables us to use a single isocenter to treat multiple lesions simultaneously with 9–11 non‐coplanar IMRT fields in a single fraction. The total IMRS treatment time is about 45 minutes, including patient setup time.

As an alternative approach to the rigid head ring, a few frameless systems have been introduced for patient positioning and target localization in intracranial SRS. One of such systems is the infrared OFLS, consisting of a customized bite block with a rigid array of fiducial markers attached. A ceiling mounted infrared camera detects the array of infrared reflective fiducials and compares with the preregistered isocenter position to provide the guidance for patient setup and tumor localization. It also continuously monitors patient's intrafraction movement during the whole treatment.^(16)^ ^,^ ^(17)^

The accuracy of optical guidance based on the fiducial readings has been tested in terms of phantom studies by Bova^(17)^ and other researchers and found to be highly precise. We have also carried out our own verification by taking measurements with a phantom mounted on the treatment couch or on a floor stand. As the phantom makes systematic shifts along lateral, longitudinal, and vertical directions, the camera readings are recorded and compared with the couch digital readings. It is found that the camera reading and couch digitals readings match with high precision and with correlation coefficient at 1.000 along all directions and also along couch rotations.^(^ ^18^ ^–^ ^22^ ^)^

However, the accuracy of using OFLS in patient treatments has not been fully addressed, and the question of the efficacy of this system on patient positioning remains unanswered.^(^ ^23^ ^–^ ^24^ ^)^ This study intends to analyze the setup accuracy for patients actually treated with OFLS localization in our clinic, and to identify factors which might affect patient setup accuracy.

## II. MATERIALS AND METHODS

**Figure 1:**
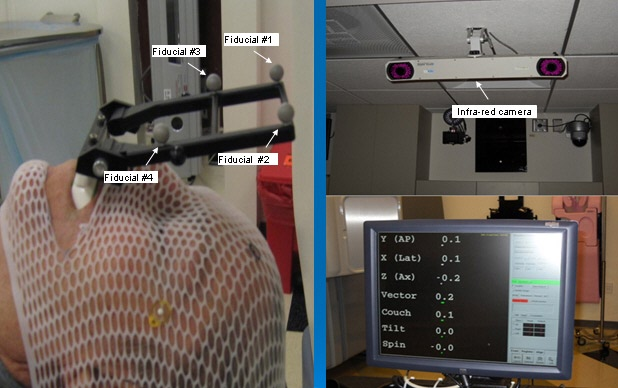
Left: patient with a customized bite block with attached fiducial array; Right: an infrared camera mounted on the ceiling (top) and the optical guidance computer (bottom) displays isocenter displacement in three translational directions in the unit of mm, and three rotational directions in degrees.

The OFLS consists of a head cushion and mask, a bite block attached with a rigid array of fiducial markers, and an in‐room ceiling mounted infrared camera system, as shown in Fig. 1. After the bite block has been customized with the bite tray filled with dental impression material, the patient is taken to the treatment room for a reseat test. This is performed first by tightening a headband of reference fiducials to the patient's head, then repeatedly taking the bite block in and out of the mouth, also with mouth open and closed, to test the robustness of the seating. Camera readings of the bite block fiducials after each repositioning are compared with the readings from the fixed reference fiducials on the headband. The maximum and the averaged test error of fiducial readings are manually recorded.

The CT simulation is done with the bite block seated in the mouth, using 512×512 pixels at 1.25 mm slice spacing and 38 cm field of view (FOV) in order to cover the whole head, including the fiducial array, head mask and its base. A magnetic resonance (MR) scan is performed without the bite block and head mask, using 26 cm FOV, 512×512 pixels, and 1–1.5 mm slice spacing.

MR and CT scans are sent either to the FastPlan or the Eclipse system (Varian Medical Systems, Palo Alto, CA) for image fusion and treatment planning, depending on whether it is a cone‐based arc plan or a MLC based IMRS treatment. FastPlan is a forward planning module dealing with arc beams using circular cones. The fusion is then performed based on anatomy matching and confirmed by a neurosurgeon. After the treatment plan is done, the planning CT images and treatment information, such as isocenter coordinates and couch angles, are sent to the optical guidance computer, where fiducials in the CT images are digitized and registered. Mean errors of fiducial positions and the isocenter displacement error from fiducial digitization are calculated, based on the known geometry of the fiducial array.

The on‐board imager (OBI) on the Trilogy linear accelerator (Varian Medical Systems) has been employed to acquire orthogonal kV images as a verification tool to validate the localization performed by OFLS. A pair of orthogonal digitally reconstructed radiographs (DRR) is created in an IMRS plan where the technique of a single isocenter is adopted; while in a SRS arc plan multiple isocenter technique is used to treat multiple lesions.

For patient head immobilization, the head rest and face mask are attached to the couch‐mount system, which provides two additional rotational adjustments. The OFLS is then used for isocenter localization, following the guidance of the camera readings of the fiducials. Two orthogonal kV images are acquired and compared with the reference planning DRR images. Radiation delivery starts only after the isocenter localization is verified by the kV images. The optical guidance is used to continuously monitor patient setup during treatment. Displacements of the actual isocenter from the preregistered isocenter position are displayed instantaneously along translational directions in 0.1 mm precision and along three rotations in 0.1° precision. If the 3D isocenter displacement is greater than 0.5 mm, the treatment is interrupted and manual adjustments are made by therapists in the treatment room. In our experience, interruption has been necessary for only a few patients.

The orthogonal kV images are used for patient setup verification only, not as an isocenter repositioning tool. Only in situations when isocenter verification is found to have a large discrepancy – greater than 2 mm in our study – as compared with the DRRs in terms of skull matching, then an isocenter shift is applied and the patient is repositioned.

**Figure 2:**
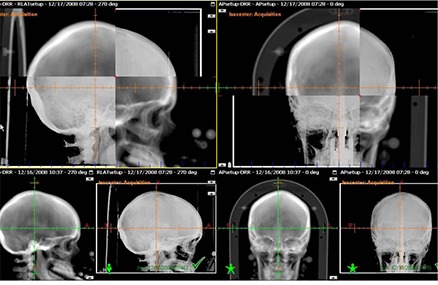
Top: Blended orthogonal images of kV setup verification at the time of treatment delivery with the reference DRRs (manual matching option is turned on in Offline Review of Varian ARIA system); Bottom: AP and lateral kV images and the reference DRRs displayed side by side.

The Offline Review in ARIA Information System version 8.1 (Varian Medical System) was employed in our retrospective study. In Offline Review, one can find the previous saved records of the numerical shifts along lateral, longitudinal, anterior‐posterior directions, and couch rotation, together with the matching kV images and DRRs. It also offers a tool for Manual Match and allows a qualified user to realign kV images with reference DRRs to assess the actual isocenter displacement. The kV images and the reference DRRs are displayed side by side, together with the blended images, as shown in Fig. 2. After adjusting the window and level of kV images, anatomic matching of the skull is carefully evaluated.

In order to identify those factors which may affect the accuracy of the OFLS system, we have collected data on patients' bite‐block reseat tests and fiducial digitization. A total of 56 patients treated in 2007 and 2008 are found to have both records available. For patients treated with five fractions, only the first fraction is included. Since the OFLS has been used with SRS and IMRS treatments, patients treated using either technique are included in this study.

## III. RESULTS

The verification results can be categorized into two distinguishing groups. The first group contains the majority, a total of 49 patients, whose kV images confirmed the OFLS localization, and treatment proceeded utilizing optical guidance. The second group contains those patients whose kV image verification detected isocenter displacement, and a clinical decision was made to apply isocenter shifts and to reposition the patient. In the latter cases, the camera readings of the fiducials were disregarded; isocenter localization was guided by the kV images and the isocenter shifts were recorded. These isocenter shifts can be reviewed and are used in our study as is. The recorded shifts in the Offline Review are found to be all greater than 2 mm in this group.

**Figure 3:**
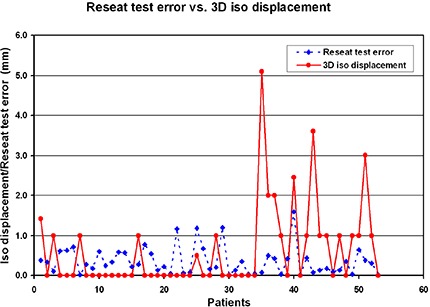
The mean error of patient reseat test is represented by the solid line; the measured 3D isocenter displacement error is represented by the dashed line.

All patients in the first group finished treatment with successful application of OFLS guidance and their records showed zero isocenter shifts. We have retrospectively studied all the patients in this group using the Manual Matching tool in Offline Review. The reported shifts are in a precision of 0.05 mm along vertical, longitudinal, and lateral couch directions, and 0.1° in couch rotation. Careful evaluation by manual matching allowed us to determine the displacement of isocenter from the intended treatment position. In our analyses, the isocenter shifts along three translational directions are combined into a 3D isodisplacement vector, defined as $E=\sqrt{vert^{2}+long^{2}+lat^{2}}$ and they are displayed as a solid polyline in Fig. 3. We have also analyzed all the available records of the reseat tests on patients' bite blocks. In Fig. 3, the blue dashed polyline represents the mean errors of the reseat tests. It shows that there is no correlation between the mean errors of patient reseating tests and the actual isocenter displacements of the treatment. The statistics of the reseating tests and the fiducial digitization are summarized in Table 1. The average error of the fiducial digitization is derived from the relative positions of the fiducials digitized on the CT, compared with that from the known geometry of the fiducial frame. This average error is then used to estimate the predicted error of the registered isocenter position from the camera system. The predicted error at isocenter would be larger if the isocenter location is further away from the fiducials. For multiple lesions, isocenter is normally selected at the center of the brain, not inside one particular lesion. It is found that the predicted error at isocenter has no direct correlation with the measured 3D isocenter displacement at treatment.

**Table 1:** Data analyses of fiducial digitization error, reseat test error, and isocenter displacement vector determined from kV images over a group of 53 patients. Three patients who failed to use OFLS for initial setup and isocenter localization are not included in the analysis.

	*Fiducial Digitization*		*Reseat Test*		*3D Isodisplacement Vector E*
	*Averaged Error*	*Predicted Error at Iso*	*Averaged Error*	*Maximum Error*	
Range (mm)	0.13 – 0.58	0.25 – 0.83	0.18 – 1.59	0.08 – 2.79	0.0 – 5.1
Median (mm)	0.27	0.44	0.28	0.51	0.0
Mean (mm)	0.28	0.46	0.36	0.63	0.61
Std (mm)	0.08	0.15	0.35	0.55	1.05
Correlation w. Vector E	0.11	0.11	0.01	0.02	1

**Figure 4:**
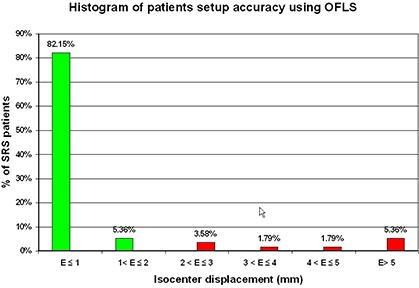
Histogram of patient setup accuracy using the OFLS intracranial radiosurgery in this study: Green represent patients who were positioned and treated with frameless localization; red represent patients who had larger isocenter displacements and were repositioned based on kV images.

Figure 4 is the histogram of the isocenter displacements of the whole patient sample. The green bar represents the patients for whom kV OBI verification was accepted and treatment proceeded following OFLS guidance; the red bar represents patients where kV‐OBI verification revealed a discrepancy between orthogonal kV images and DRRs of the planning CT and isocenter was corrected by auto couch shifts or in‐room rotation adjustment. Retrospective study using manual matching of kV images with DRRs found 82.15% of patients with isocenter displacement E≤1mm; 5.36% of patients with isocenter displacement 1<E≤2mm. Therefore, 49 patients (87.5% in this sample group) have been treated under optical guidance using OFLS. Quantitative analysis of the 3D isocenter displacements has found that patient setup accuracy had a maximum error of 2 mm, with a median of 0 mm, and a mean of 0.37 mm. This is listed in Table 2.

**Table 2:** Breakdown of 3D isodisplacements determined from kV images of 49 patients listed in who finished intracranial radiosurgery under successful optical guidance using OFLS.

*3D Iso Displacement (mm)*	*# of Patients*	*% of Patients*
0.0	33	67%
0.5	1	2%
1.0	12	25%
1.4	1	2%
2.0	2	4%
Mean: 0.37 mm	Total patients: 49	Total %: 100

This mean error of 0.37 mm is on the same order of the uncertainty of the fiducial digitization, which has a mean digitization error of 0.28 mm and mean predicted isocenter error of 0.46 mm. The fiducial digitization error is mainly caused by the image resolution and patient movement during the CT scan.

For the remaining six patients (12.5% in this group), OFLS optical guidance failed to provide accurate localization due to issues related to the patient or due to mechanical issues related to the bite block. In addition, there are three patients whose bite block could not be used, presumably due to movement of the fiducial array after the CT scan was completed. For these patients, the isocenter was localized by the OBI system only. We, therefore, assigned a value of E>5mm to this subgroup, in order to more faithfully reflect the probability of success and failure in our analysis. This subgroup of patients is represented by the last vertical bar in Fig. 4.

## IV. DISCUSSION

Retrospective analysis of the setup accuracy using OFLS for actual patient treatment has been carried out in this study. Our study confirms the efficacy of the optical guided frameless system for intracranial stereotactic radiosurgery. For patients who completed treatment under the optical guidance, the 3D isocenter localization is found to have a maximum error of 2 mm, average of 0.37 mm, and a median of 0.0 mm in our patient sample.

We have used the OFLS for patient immobilization and isocenter localization in both cone‐based SRS and MLC‐based IMRS techniques. In our practice, we have become increasingly aware of the importance of using kV imaging as a verification tool together with the OFLS optical guidance for isocenter localization. Therefore, the setup fields are always added into the IMRS plan so that an orthogonal pair of DRRs can be generated. For the cone‐based SRS plan, we have learned to add the setup fields outside FastPlan after exporting the cone‐based SRS plan to the Eclipse system.

The treatment isocenter localization is done utilizing the optical guidance system by adjusting couch positions and rotations along right‐left, cranial‐caudal axes and anterior‐posterior axes. The kV‐OBI is meant to be used as a verification tool only. For the 49 patients (87.5%) in this study, no isocenter corrections were applied based on the OBI images. The kV‐DRR matching was performed on the OBI workstation in the treatment control room prior to treatment. Having therapist, physicist, and radiation oncologist review the image matching together may reduce, but can not completely eliminate, the uncertainty introduced by the observer. It is found that the approved image matching of patients in this study has a standard deviation of 0.37 mm and up to a maximum of 2 mm uncertainty.

**Figure 5:**
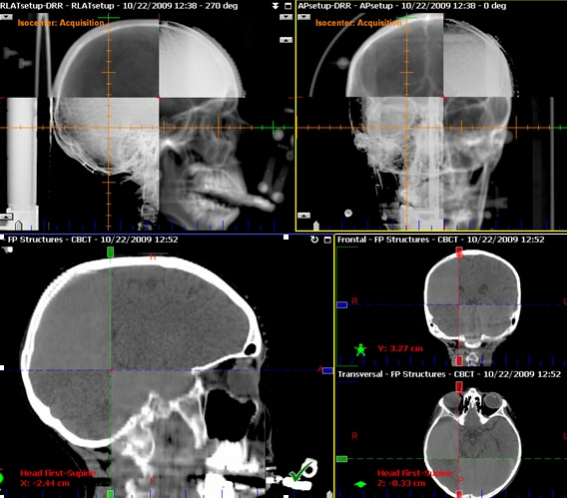
Comparison of kV‐DRR (top) and 3D‐CBCT (bottom) matching of a recently‐treated patient.

We have used kV‐OBI in place of CBCT as a verification tool for the majority of the patients to minimize the length of time a patient must stay in the treatment position. Extending the treatment time may reduce patient's tolerance for holding the bite block in place. This is undesirable as the mouth movement, muscle contractions, and discomfort may affect the treatment accuracy. Also in intracranial cases, as opposed to other anatomical areas, the relative positions of the internal anatomy and the bony skull are unlikely to change from simulation to treatment. Therefore, the skull‐matching based on the orthogonal pair of kV‐DRR images was appropriate for clinical evaluation.^(25)^ We have acquired both kV‐OBI images and CBCT for a few patients and performed matching and review. We found that the two techniques were comparable in the intracranial cases. As an example, the fusion results from the matching of 2D‐kV (top) and 3D‐CBCT (bottom) is shown in Fig. 5, which is from a recently‐treated patient. In the earlier version of ARIA used in 2007 and 2008, 3D‐CBCT matching could not be saved.

For seven patients (12.5%) in this study, repositioning was needed based on kV‐DRR matching. The camera is calibrated to the mechanic isocenter of the Linac and verified before the treatment. The kV imaging isocenter was calibrated to the radiation isocenter with an accuracy of less than 1 mm. Weekly verification using orthogonal kV images and CBCT of a cubic phantom is done in the clinic, and the overall discrepancy is found to be around 1 mm between lasers and imaging isocenter. This is consistent with the general findings by other groups.^(^ ^26^ ^–^ ^27^ ^)^

CBCT does provide more information on soft tissue and better assesses rotation along the cranial‐caudal axis. With improved CBCT techniques and better image recording and evaluation tools in ARIA v8.6, we are now shifting to using CBCT as a verification tool in intracranial SRS cases.

We have identified several patient related issues, such as mouth breathing, mask contraction, or changes in the biting, which may lead to the failure of the optical guidance using OFLS. This is resolved by obtaining a new CT simulation, or continuing treatment by using kV images for isocenter localization. In the latter case, the bite block is kept in the mouth to maintain patient setup congruence, but camera readings are disregarded as repositioning the patient would disqualify the camera readings. This is a clinical decision and requires a physician's approval following a review of the kV images and DRRs with the therapist and physicist.

Contrary to the general belief that the result from the reseat test can be used as an indication of the reliability of patient setup accuracy, our study has found that there was no correlation between the mean error of a patient's reseat test and the 3D isocenter displacement at treatment. This may arise from the fact that the patients' treatments were scheduled several days after the CT simulation. Reseating test still serves the purpose of filtering out patients with large reseating test errors before the radiosurgery planning is started. As large reseat test error often indicates a biting problem, it may affect the reproducibility of patient setup when using the OFLS. We encountered a few such cases and a different treatment technique, such as IMRT, was offered in combination with using different devices for immobilization.

## V. CONCLUSIONS

The IMRS treatment employed the single isocenter, single fraction approach to treat multiple brain lesions simultaneously. Using the OFLS for patient setup and isocenter localization in the IMRS treatment makes the above approach possible.^(^ ^12^ ^,^ ^14^ ^–^ ^15^ ^)^

Our study further concludes that orthogonal kV imaging or CBCT verification is a necessity to ensure the efficacy of patient setup using the OFLS. In our data sample of 56 patients, 49 (87.5%) patients have been treated under optical guidance using this system.

The planning target volume (PTV) in the IMRS planning primarily depends on the clinical tumor volume (CTV), and is used to select appropriate beam sizes and beam arrangements, as in 3D conformal and IMRT treatment techniques. We have been adding either none or a small margin of 1–2 mm to the CTV to form the PTV. Based on our findings from this study, we are now routinely adding a 2 mm margin to CTV to create the PTV, as the study concludes that a 2 mm margin should be used to account for actual patient setup accuracy using the OFLS.
